# Long non‐coding RNA LINC00526 represses glioma progression via forming a double negative feedback loop with AXL

**DOI:** 10.1111/jcmm.14435

**Published:** 2019-06-26

**Authors:** Jian Yan, Chunhua Xu, Youping Li, Bin Tang, Shenhao Xie, Tao Hong, Erming Zeng

**Affiliations:** ^1^ Department of Neurosurgery The First Affiliated Hospital of Nanchang University Nanchang China

**Keywords:** AXL, feedback loop, glioma, long non‐coding RNA, progression

## Abstract

Glioma is the most common primary intracranial carcinoma with extremely poor prognosis. The significances of long non‐coding RNA (lncRNA) involved in glioma have been started revealed. However, the expression, roles and molecular mechanisms of most lncRNAs in glioma are still unknown. In this study, we identified a novel lncRNA LINC00526, which is significantly low expressed in glioma. Low expression of LINC00526 is correlated with aggravation and poor survival in glioma. Functional assays revealed that ectopic expression of LINC00526 inhibits glioma cell proliferation, migration, and invasion. LINC00526 silencing promotes glioma cell proliferation, migration and invasion. Mechanistically, we found that LINC00526 directly interacts with EZH2, represses the binding of EZH2 to *AXL* promoter, attenuates the transcriptional activating roles of EZH2 on *AXL*, and therefore represses *AXL* expression. Via repressing AXL, LINC00526 further represses PI3K/Akt/NF‐κB signalling. Intriguingly, we identified that NFKB1 and NFKB2 directly binds *LINC00526* promoter and represses *LINC00526* transcription. We further found that via activating NF‐κB signalling, AXL represses *LINC00526* transcription. Therefore, LINC00526/EZH2/AXL/PI3K/Akt/NF‐κB form a feedback loop in glioma. Analysis of the TCGA data revealed that the expression of LINC00526 is inversely correlated with that of AXL in glioma tissues. In addition, functional rescue assays revealed that the tumour suppressive roles of LINC00526 are dependent on the negative regulation of AXL. Collectively, our data identified LINC00526 as a tumour suppressor in glioma via forming a double negative feedback loop with AXL. Our data also suggested LINC00526 as a potential prognostic biomarker and therapeutic candidate for glioma.

## 1 INTRODUCTION

Glioma is the most prevalent type of intracranial neoplasm with high morbidity and mortality.^1^ Although multiple therapeutic strategies have emerged, including surgical resection, radiotherapy, chemotherapy and so on, the outcomes of glioma patients are still very poor due to the excessive aggressive growth of glioma and resistance to conventional therapies.^2^, ^3^ Therefore, revealing the in‐depth molecular pathogenesis of glioma is of great importance to develop efficient therapies for glioma.

Accumulating genome and transcriptome high‐throughput sequencings have confirmed that most of human genome is transcribed for non‐coding RNAs.^4^, ^5^ For example, Iyer et al identified 58,648 genes transcribing for long non‐coding RNAs, and only 21,313 genes encoding for proteins in human cells.^6^ Long non‐coding RNA (lncRNA) is a class of transcripts with limited protein‐coding potential and more than 200 nucleotides in length.^7^ Increasing evidences have demonstrated that many lncRNAs are frequently dysregulated in numerous diseases, including cancers.^8^, ^9^, ^10^ Among these dysregulated lncRNAs, several lncRNAs are further verified to be potential diagnostic and prognostic biomarkers for a variety of cancers.^11^, ^12^, ^13^ Furthermore, many lncRNAs have important roles in various physiological and pathological processes.^14^, ^15^, ^16^ As to cancers, numerous lncRNAs have been shown to influence cell proliferation, cell cycle, cell apoptosis, cell senescence, cell migration and invasion, drug‐resistance, in vivo tumour growth and metastasis and so on.^17^, ^18^, ^19^, ^20^ Therefore, several lncRNAs were identified to be potential therapeutic targets for various cancers.^21^ LncRNAs have various and complex regulatory roles in control gene expression, particular for the expressions of critical oncogenes and tumour suppressors.^22^, ^23^, ^24^ LncRNAs modulate gene expression at transcriptional and post‐transcriptional levels via interacting with DNAs, proteins, mRNAs and/or microRNAs in different cellular contexts.^25^, ^26^, ^27^

The expression, roles and clinical significances of several lncRNAs in glioma have also been reported.^28^ Fu et al reported that lncRNA PVT1 is highly expressed in glioma and facilitates glioma tumorigenesis and progression.^29^ Wang et al revealed that lncRNA‐135528 inhibits glioma progression.^30^ Liu et al found that lncRNA LINC00470 promoted glioma progression.^31^ Xu et al identified that lncRNA AC003092.1 promotes glioma temozolomide chemosensitivity.^32^ Although several lncRNAs have been investigated in glioma, the expression, roles and functional mechanisms of most other lncRNAs in glioma are still unknown.

In a previous report, Zhou et al compared the differentially expressed lncRNAs in 14 glioma tissues and five normal tissues using lncRNA microarray analysis.^33^ Among these differentially expressed lncRNAs, we noted lncRNA LINC00526, which is markedly down‐regulated in glioma tissues. LINC00526 locates at chromosome 18p11.31, with 1322 nucleotides in length. Open reading frame (ORF) Finder from NCBI (https://www.ncbi.nlm.nih.gov/orffinder/) and Coding Potential Calculator (http://cpc.cbi.pku.edu.cn/programs/run_cpc.jsp) both confirmed that LINC00526 has no protein coding capability. In this study, we further investigated the express pattern of LINC00526 in larger glioma tissue samples, analysed its correlation with clinical features and prognosis, confirmed its biological roles in glioma progression and explored the potential molecular mechanisms mediating the dysregulation of LINC00526 in glioma and the mechanisms responding for the roles of LINC00526 in glioma.

## 2 MATERIALS AND METHODS

### 2.1 Tissue samples

Human glioma tissues were obtained from 41 glioma patients who received surgery at the first affiliated hospital of Nanchang university (Nanchang, Jiangxi, China). Normal brain tissues were obtained from 11 patients who received brain tissue resection for craniocerebral injury at the first affiliated hospital of Nanchang university (Nanchang, Jiangxi, China). All tissues were pathologically diagnosed. Tissue samples were preserved at −80°C. The uses of tissue samples were reviewed and approved by the ethics committee of the first affiliated hospital of Nanchang university (Nanchang, Jiangxi, China). Written informed consents were obtained from all patients.

### 2.2 Cell culture and treatment

Human normal glia cell line NHA, and glioma cell lines LN18, U87, U251 and U138 were obtained from cell banks of the Chinese Academy of Sciences (Shanghai, China). NHA, LN‐18 and U251 cells were cultured in Dulbecco's Modified Eagle's Medium (DMEM) (Invitrogen, Carlsbad, CA, USA) with 10% foetal bovine serum (FBS) (Invitrogen). U87 and U138 cells were cultured in Eagle's Minimum Essential Medium (MEM) (Invitrogen) with 10% FBS. All the cells were maintained in an incubator containing 5% CO_2_ at 37°C. Where indicated, glioma cells were treated with 10 μM Andrographolide (MedChemExpress, Monmouth Junction, NJ, USA) or 1 μM R428 (MedChemExpress) for indicated time.

### 2.3 RNA extraction, reverse transcription and quantitative real‐time polymerase chain reaction (qRT‐PCR)

Total RNA was extracted from indicated tissues or cells using the RNAiso Plus (Takara, Dalian, China), followed by treatment with DNase I (Takara) to remove genomic DNA. The extracted RNA was further used for reverse transcription with PrimeScript™ II 1st Strand cDNA Synthesis Kit (Takara). The generated cDNA was then used for quantitative real‐time polymerase chain reaction (qRT‐PCR) with SYBR^®^ Premix Ex Taq™ II kit (Takara) on ABI7500 Real‐Time PCR System (Applied Biosystems, Foster City, CA, USA) following the standard SYBR Green protocol. The primers’ sequences were as follows: for LINC00526, 5′‐TTCAGGCTTCTGGGTCTC‐3′ (sense) and 5′‐TTCACGGTTGGTATTTCGG‐3′ (antisense); for AXL, 5′‐CACCTCCCTGCAGCTTTC‐3′ (sense) and 5′‐CTCAGGTTGAAGGGGGTG‐3′ (antisense)^34^; and for GAPDH, 5′‐GGTCTCCTCTGACTTCAACA‐3′ (sense) and 5′‐GTGAGGGTCTCTCTCTTCCT‐3′ (antisense).^35^ GAPDH was employed as an endogenous control. The quantification of the expression of RNAs was calculated by the 2^−ΔΔCt^ method.

### 2.4 Plasmids construction

LINC00526 full‐length sequences were PCR‐amplified by the *PfuUltra* II Fusion HS DNA Polymerase (Agilent Technologies, Santa Clara, CA, USA) and the primers 5′‐CGGGATCCGCGGACTCCGCGGACAAG‐3′ (sense) and 5′‐GGAATTCCAAAATGCATCTTGTTTATTTGGC‐3′ (antisense). Then, the PCR products were cloned into the BamH I and EcoR I sites of pcDNA^TM^3.1(+) (Invitrogen) and pSPT19 (Roche, Mannheim, Germany) plasmids, named as pcDNA‐LINC00526 and pSPT19‐LINC00526, respectively. The cDNA oligonucleotides silencing LINC00526 were synthesized by GenePharma (Shanghai, China) and inserted into the GenePharma SuperSilencing^TM^ shRNA expression plasmid pGPU6/Neo. The shRNAs target sites were 5′‐GCTCAATGTCTCATAGCTACA‐3′ (shLinc‐1) and 5′‐GGTCCTCCAAGATGAGCTTAA‐3′ (shLinc‐2). AXL ORF expression plasmid (Catalog EX‐Z7835‐M68) was purchased from GeneCopoeia (Guangzhou, China).

### 2.5 Small interfering RNA (siRNA) synthesis and transfection

AXL specific and control Stealth siRNAs (siRNA ID HSS100897) were purchased from Thermo Fisher Scientific (Waltham, MA, USA). The transfection of plasmids and siRNAs was carried out by the Lipofectamine 3000 (Invitrogen) following the protocol.

### 2.6 Stable cell lines construction

To obtain LINC00526 stably ectopically expressed glioma cells, pcDNA‐LINC00526 or pcDNA empty plasmids were transfected into U87 and U251 cells. The cells were treated with neomycin 48 hours after transfection for 4 weeks to select LINC00526 stably ectopically expressed cells. To obtain LINC00526 stably silenced glioma cells, shLinc‐1 or shLinc‐2 was transfected into U87 and U251 cells. The cells were treated with neomycin 48 hours after transfection for 4 weeks to select LINC00526 stably silenced cells. To obtain LINC00526 and AXL concurrently stably ectopically expressed glioma cells, AXL ORF expression plasmid (Catalog EX‐Z7835‐M68) was transfected into LINC00526 stably ectopically expressed U87 cells. The cells were treated with puromycin 48 hours after transfection for 4 weeks to select LINC00526 and AXL concurrently stably ectopically expressed cells.

### 2.7 Cell proliferation assays

Cell proliferation was evaluated by Cell Counting Kit‐8 (CCK‐8) and Ethynyl deoxyuridine (EdU) incorporation assays. For CCK‐8 assay, indicated glioma cells were seeded into 96‐well plates at a density of 2000 cells per well. After culture for indicated time, 10 μL CCK‐8 solution (Dojindo, Kumamoto, Japan) was added into per well. After incubation for 1 hour, the absorbance at 450 nm was measured to indicate cell proliferation rate. EdU incorporation assay was performed using the EdU kit (RiboBio, Guangzhou, China) in accordance with the protocol. The results were acquired by the Zeiss Photomicroscope (Carl Zeiss, Oberkochen, Germany) and analysed by counting at least five random fields.

### 2.8 Cell migration and invasion assays

Transwell migration and invasion assays were undertaken to evaluate the cell migration and invasion potential. Indicated glioma cells resuspended in serum‐free medium were seeded into the upper chamber of 24‐well inserts with 8 μm pores (Millipore, Billerica, MA, USA). For invasion assay, the upper chamber was pre‐coated with Matrigel (BD Biosciences, San Jose, CA, USA). Medium containing 10% FBS was added to lower chamber. After culture at 37°C for 48 hours, the cells remaining on the upper chamber were scraped off, and while those on the lower side of chamber were fixed, stained and observed using a Zeiss Photomicroscope. The results were analysed by counting at least five random fields.

### 2.9 Cytoplasmic and nuclear RNA purification

Cytoplasmic and nuclear RNA were purified from U87 cells using the Cytoplasmic & Nuclear RNA Purification Kit (Norgen, Belmont, CA, USA) following the instruction. After purification, the RNA was used for reverse transcription and qRT‐PCR as above described. U6 was employed as nuclear control, and while β‐actin was employed as cytoplasmic control. The primers used were as follows: for LINC00526, 5′‐TTCAGGCTTCTGGGTCTC‐3′ (sense) and 5′‐TTCACGGTTGGTATTTCGG‐3′ (antisense); for U6, 5′‐GCTTCGGCAGCACATATACTAAAAT‐3′ (sense) and 5′‐CGCTTCACGAATTTGCGTGTCAT‐3′ (antisense)^35^ and for β‐actin, 5′‐GGGAAATCGTGCGTGACATTAAG‐3′ (sense) and 5′‐TGTGTTGGCGTACAGGTCTTTG‐3′ (antisense).^35^

### 2.10 RNA immunoprecipitation (RIP) assay

RNA immunoprecipitation (RIP) assay was conducted using U87 cells with the Magna RIP^TM^ RNA‐Binding Protein Immunoprecipitation Kit (Millipore) and EZH2 specific primary antibody (Millipore) in accordance with the protocol. The enriched RNA was used for reverse transcription and qRT‐PCR as above described. The primers used were as follows: for LINC00526, 5′‐TTCAGGCTTCTGGGTCTC‐3′ (sense) and 5′‐TTCACGGTTGGTATTTCGG‐3′ (antisense); for HOTAIR, 5′‐TTCCACAGACCAACACCC‐3′ (sense) and 5′‐CTAAATCCGTTCCATTCCA‐3′ (antisense) and for GAPDH, 5′‐GGTCTCCTCTGACTTCAACA‐3′ (sense) and 5′‐GTGAGGGTCTCTCTCTTCCT‐3′ (antisense).^35^

### 2.11 RNA pull‐down assay

LINC00526 full‐length sequences were in vitro transcribed and biotin‐labelled from pSPT19‐LINC00526 with the Biotin RNA Labeling Mix (Roche) and T7 RNA polymerase (Roche) following their protocols. LINC00526 antisense RNA was in vitro transcribed and biotin‐labelled from pSPT19‐LINC00526 with the Biotin RNA Labeling Mix (Roche) and Sp6 RNA polymerase (Roche) following their protocols. After treatment with DNase I (Takara), the in vitro transcribed RNA was purified by the RNeasy Mini Kit (Qiagen, Valencia, CA, USA). Next, 3 µg of purified biotinylated RNA was incubated with 1 mg of U87 or U251 whole‐cell lysates at 25°C for 1 hour. Then, the complexes were isolated by streptavidin agarose beads (Invitrogen). The enriched proteins in the complexes were measured by Western blot.

### 2.12 Western blot

Total cell lysates were isolated from indicated glioma cells by the RIPA lysis buffer (Beyotime, Shanghai, China). Identical quantities of proteins were separated by sodium dodecyl sulfate‐polyacrylamide gel electrophoresis (SDS‐PAGE) and transferred into PVDF membrane (Millipore). After being blocked with 5% non‐fat milk, the blots were further incubated with primary antibodies against AXL (Cell Signaling Technology, Danvers, MA, USA), phosphorylated Akt (p‐Akt) (Cell Signaling Technology), Akt (Cell Signaling Technology), phosphorylated IκBα (p‐IκBα) (Cell Signaling Technology), EZH2 (Millipore) or GAPDH (Proteintech, Rosemont, IL, USA). After three washes, the blots were incubated with IRdye 700‐conjugated goat anti‐mouse IgG (Thermo Fisher Scientific) or IRdye 800‐conjugated goat anti‐rabbit IgG (Thermo Fisher Scientific). After three washes, the blots were detected by Odyssey Infrared Scanner (Li‐Cor, Lincoln, NE, USA).

### 2.13 Chromatin immunoprecipitation (ChIP) assay

ChIP assay was undertaken using indicated glioma cells with the EZ‐Magna ChIP™ A/G Chromatin Immunoprecipitation Kit (Millipore) and EZH2 specific primary antibody (Millipore), NFKB1 specific primary antibody (Sigma‐Aldrich, Saint Louis, MO, USA) or NFKB2 specific primary antibody (Sigma‐Aldrich) following the protocol. The enriched DNA was measured by qRT‐PCR as above described. The primers used were as follows: for *LINC00526* promoter, 5′‐CCCGACTGTTTCCTACCC‐3′ (sense) and 5′‐GCCTCCCGACTTTTTGATG‐3′ (antisense); for *AXL* promoter, 5′‐GAGTGAGGGGGAATGAAGG‐3′ (sense) and 5′‐TCTGGGCTCTGTGTCTGGT‐3′ (antisense).

### 2.14 Statistical analysis

GraphPad Prism 5.0 was used to carry out statistical analyses. For comparisons, Kruskal‐Wallis test followed by Dunn's multiple comparison test, log‐rank test, Student's *t* test, one‐way ANOVA followed by Dunnett's multiple comparison test, or Pearson correlation analysis were carried out as indicated. *P* < 0.05 was considered as statistically significant.

## 3 RESULTS

### 3.1 LINC00526 was lowly expressed in glioma

**Figure 1:**
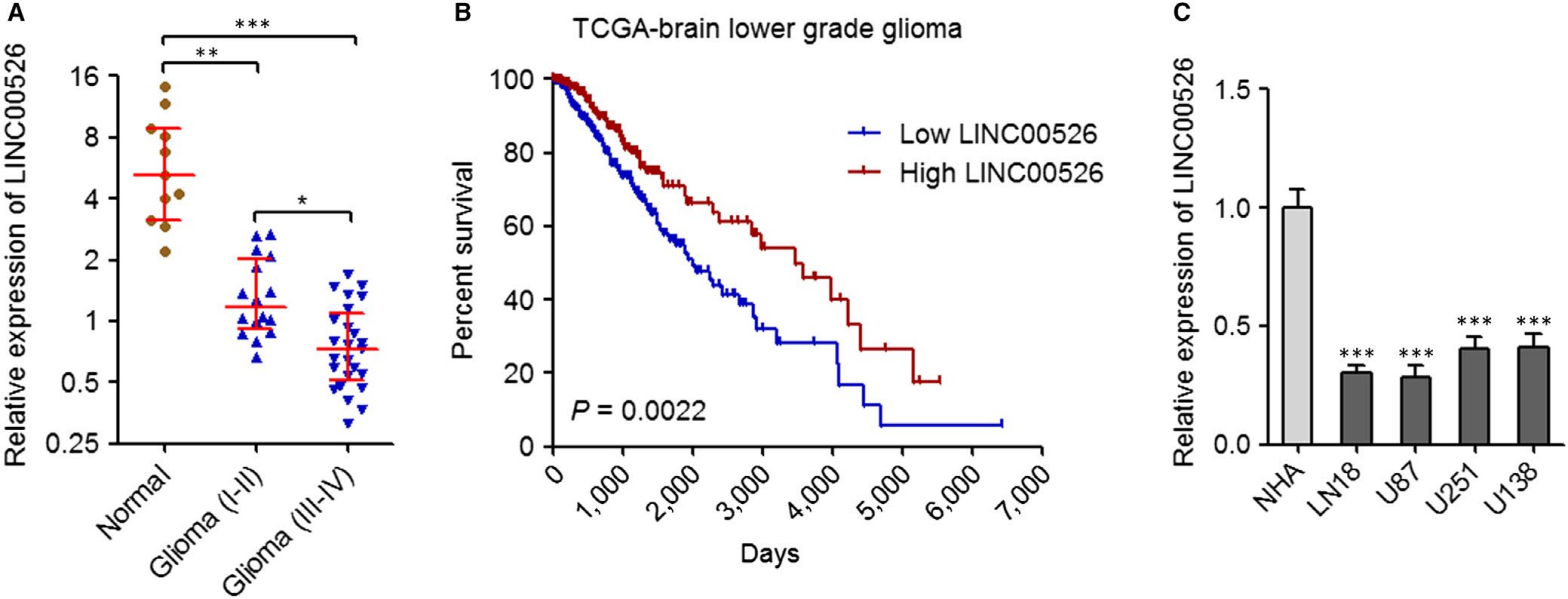
LINC00526 was lowly expressed in glioma. A, LINC00526 expression levels in 25 glioma tissues (III‐VI), 16 glioma tissues (I‐II) and 11 normal tissues were measured by qRT‐PCR. Results are shown as median with interquartile range. *P < 0.05, **P < 0.01, ***P < 0.001, Kruskal‐Wallis test followed by Dunn's multiple comparison test. B, Kaplan‐Meier survival analyses of the correlation between LINC00526 expression level and overall survival of brain lower grade glioma patients (*n* = 510) from TCGA data. LINC00526 median expression level was used as cut‐off. *P* = 0.0022, log‐rank test. C, LINC00526 expression levels in normal glia cell line NHA and glioma cell lines LN18, U87, U251 and U138 were measured by qRT‐PCR. Results are shown as mean ± SD of three independent experiments. ***P < 0.001, one‐way ANOVA followed by Dunnett's multiple comparison test

We first measured LINC00526 expression level in 41 glioma tissues and 11 normal tissues. As shown in Figure 1A, LINC00526 was obviously lowly expressed in glioma tissues compared with normal tissues. Moreover, LINC00526 was further decreased in aggravated glioma tissues (Figure 1A). The Cancer Genome Atlas (TCGA) data revealed that lower expression of LINC00526 was associated with shorter survival time in brain low grade glioma patients (Figure 1B). Furthermore, LINC00526 expression level in normal glia cell line NHA and glioma cell lines LN18, U87, U251 and U138 was measured. The results showed that LINC00526 was lowly expressed in glioma cell lines compared with normal glia cell line (Figure 1C). Thus, these results indicated that LINC00526 was lowly expressed in glioma. Low expression of LINC00526 was associated with the aggravation and poor prognosis of glioma.

### 3.2 Ectopic expression of LINC00526 inhibited the proliferation, migration and invasion of glioma cells

**Figure 2:**
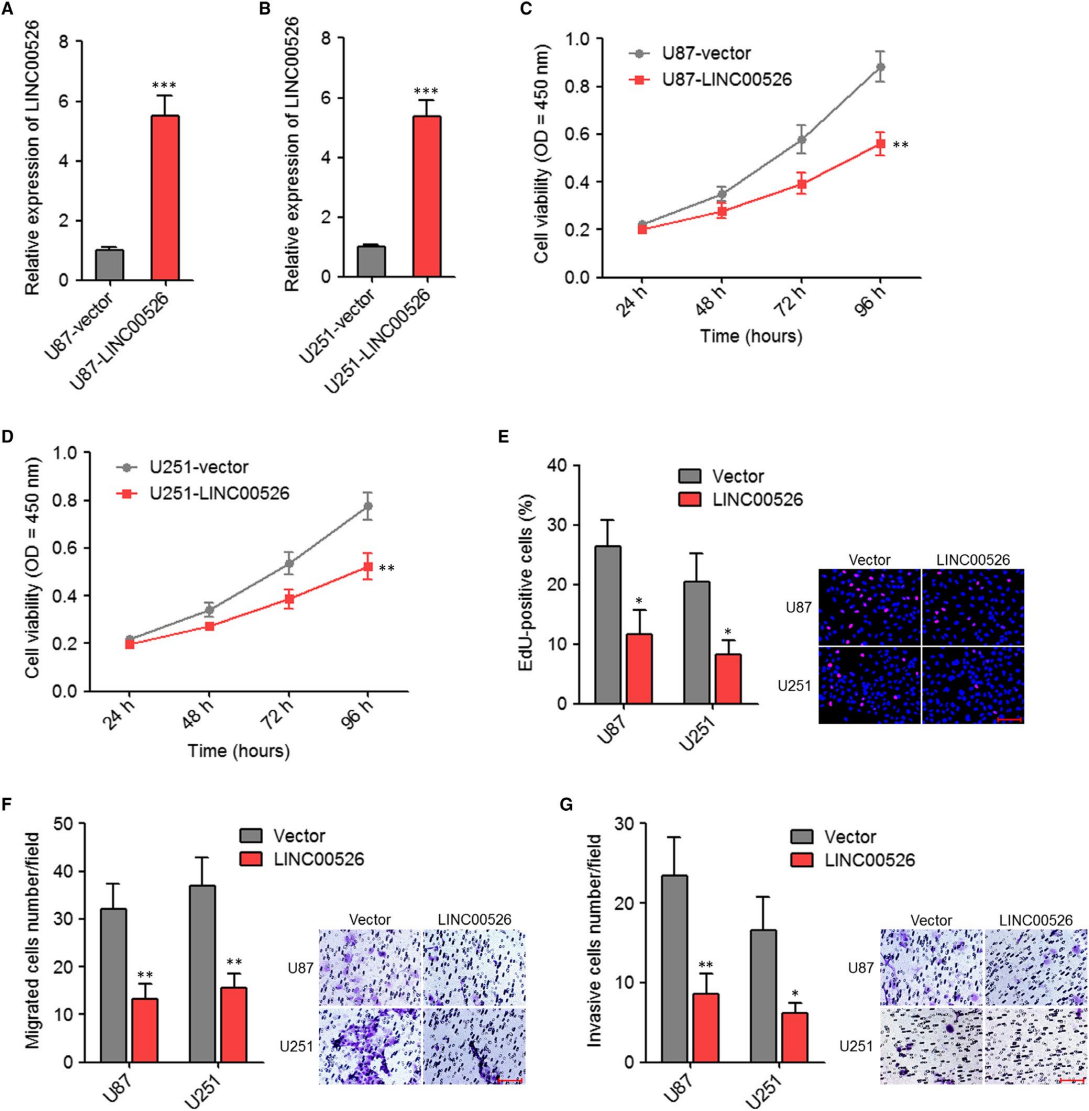
Ectopic expression of LINC00526 repressed glioma cell proliferation, migration and invasion. A, LINC00526 expression levels in LINC00526 stably ectopically expressed and control U87 cells were measured by qRT‐PCR. B, LINC00526 expression levels in LINC00526 stably ectopically expressed and control U251 cells were measured by qRT‐PCR. C, Cell proliferation rates of LINC00526 stably ectopically expressed and control U87 cells were measured by CCK‐8 assays. OD values in 450 nm were detected to indicate cell proliferation. D, Cell proliferation rates of LINC00526 stably ectopically expressed and control U251 cells were measured by CCK‐8 assays. OD values in 450 nm were detected to indicate cell proliferation. E, Cell proliferation of LINC00526 stably ectopically expressed and control U87 and U251 cells were measured by EdU incorporation assays. Scale bars, 100 µm. F, Cell migration of LINC00526 stably ectopically expressed and control U87 and U251 cells were measured by transwell migration assays. Scale bars, 100 µm. G, Cell invasion of LINC00526 stably ectopically expressed and control U87 and U251 cells were measured by transwell invasion assays. Scale bars, 100 µm. Results are shown as mean ± SD of three independent experiments. *P < 0.05, **P < 0.01, ***P < 0.001, Student's *t* test

To determine the influences of LINC00526 on glioma cells, we stably ectopically expressed LINC00526 in U87 and U251 cells (Figure 2A,2). CCK‐8 assays revealed that ectopic expression of LINC00526 obviously decreased the proliferation rate of U87 and U251 cells (Figure 2C,2). Moreover, EdU incorporation assays further supported the repressive roles of ectopic expression of LINC00526 on the proliferation of U87 and U251 cells (Figure 2E). Transwell migration assays revealed that ectopic expression of LINC00526 significantly decreased the migration of U87 and U251 cells (Figure 2F). Transwell invasion assays revealed that ectopic expression of LINC00526 significantly decreased the invasion of U87 and U251 cells (Figure 2G). Thus, these results indicated that ectopic expression of LINC00526 inhibited the proliferation, migration and invasion of glioma cells.

### 3.3 LINC00526 silencing promoted the proliferation, migration and invasion of glioma cells

**Figure 3:**
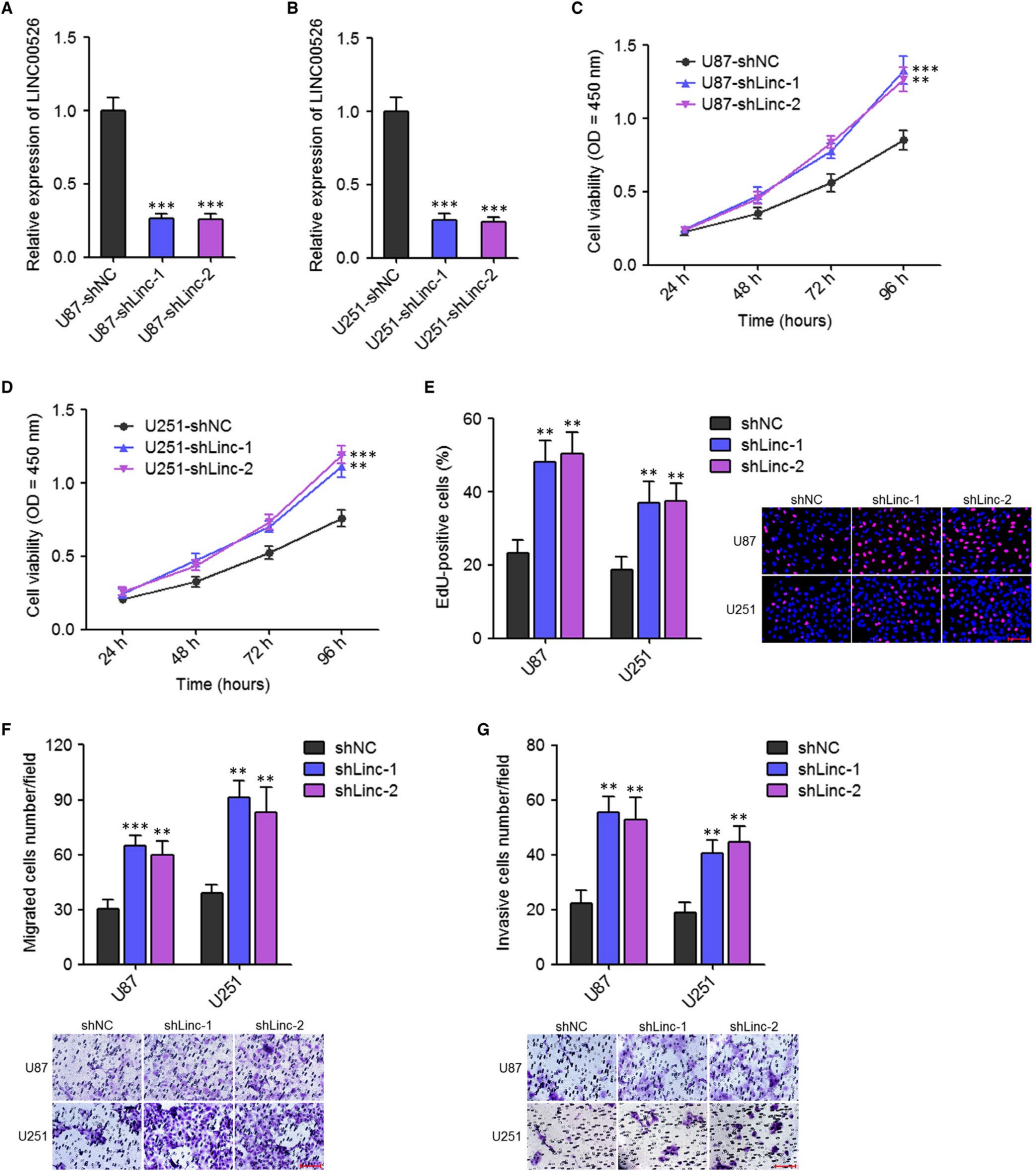
LINC00526 silencing promoted glioma cell proliferation, migration and invasion. A, LINC00526 expression levels in LINC00526 stably silenced and control U87 cells were measured by qRT‐PCR. B, LINC00526 expression levels in LINC00526 stably silenced and control U251 cells were measured by qRT‐PCR. C, Cell proliferation rates of LINC00526 stably silenced and control U87 cells were measured by CCK‐8 assays. OD values in 450 nm were detected to indicate cell proliferation. D, Cell proliferation rates of LINC00526 stably silenced and control U251 cells were measured by CCK‐8 assays. OD values in 450 nm were detected to indicate cell proliferation. E, Cell proliferation of LINC00526 stably silenced and control U87 and U251 cells were measured by EdU incorporation assays. Scale bars, 100 µm. F, Cell migration of LINC00526 stably silenced and control U87 and U251 cells were measured by transwell migration assays. Scale bars, 100 µm. G, Cell invasion of LINC00526 stably silenced and control U87 and U251 cells were measured by transwell invasion assays. Scale bars, 100 µm. Results are shown as mean ± SD of three independent experiments. **P < 0.01, ***P < 0.001, one‐way ANOVA followed by Dunnett's multiple comparison test

To further confirm the tumour suppressive roles of LINC00526 in glioma, we stably silenced the expression of LINC00526 in U87 and U251 cells using two independent LINC00526 specific shRNAs (Figure 3A,3). CCK‐8 assays revealed that silencing of LINC00526 obviously increased the proliferation rate of U87 and U251 cells (Figure 3C,3). Moreover, EdU incorporation assays further supported the roles of LINC00526 silencing in promoting the proliferation of U87 and U251 cells (Figure 3E). Transwell migration assays revealed that LINC00526 silencing significantly promoted the migration of U87 and U251 cells (Figure 3F). Transwell invasion assays revealed that LINC00526 silencing significantly promoted the invasion of U87 and U251 cells (Figure 3G). Thus, these results indicated that LINC00526 silencing promoted the proliferation, migration and invasion of glioma cells.

### 3.4 The expression of LINC00526 was inversely correlated with that of AXL in glioma tissues

**Figure 4:**
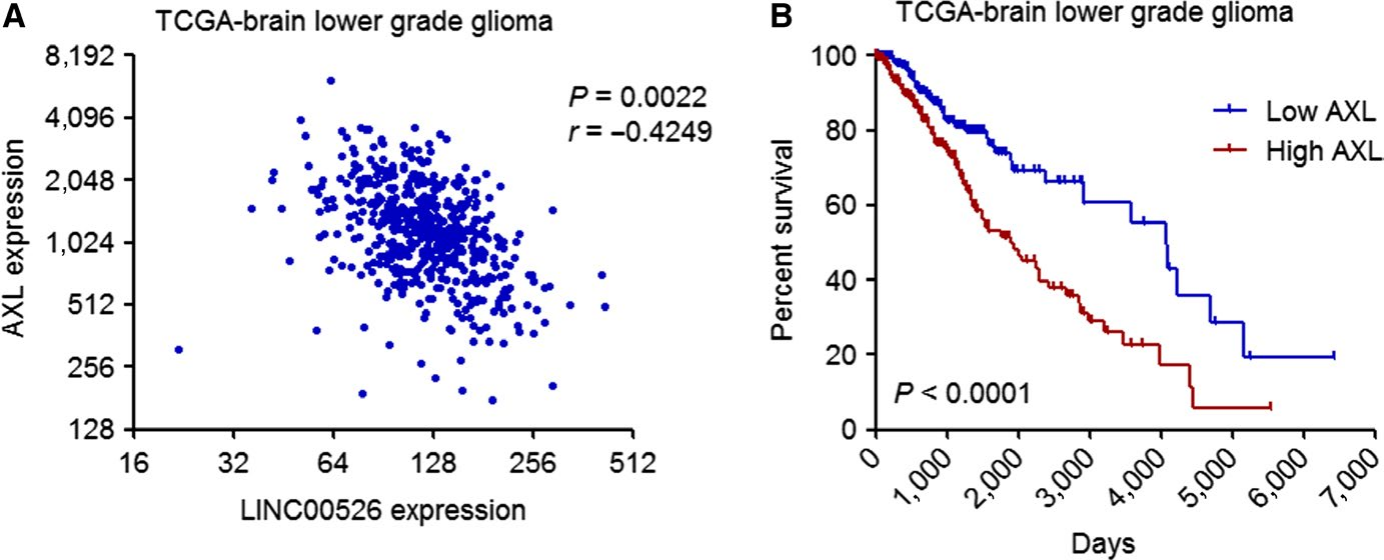
The expression levels of AXL were inversely correlated with that of LINC00526 in glioma tissues. A, The correlations between AXL expression level and LINC00526 expression level in brain lower grade glioma tissues (n = 510) from TCGA data. *r* = −0.4249, *P* = 0.0022, Pearson correlation analysis. B, Kaplan‐Meier survival analyses of the correlation between AXL expression level and overall survival of brain lower grade glioma patients (n = 510) from TCGA data. AXL median expression level was used as cut‐off. *P* < 0.0001, log‐rank test

To investigate the potential mechanisms mediating the tumour suppressive roles of LINC00526 in glioma, we analysed the expression correlations between LINC00526 and mRNAs in glioma tissues using TCGA data. Among the mRNAs whose expressions are correlated with LINC00526, we noted AXL, which is a receptor tyrosine kinase and has critical oncogenic roles in many cancers including glioma.^36^, ^37^ As shown in Figure 4A, AXL mRNA expression was significantly inversely correlated with that of LINC00526 in glioma tissues (*r* = −0.4249, *P* = 0.0022). In contrast to LINC00526, higher expression of AXL was associated with shorter survival time in glioma patients (Figure 4B).

### 3.5 LINC00526 repressed *AXL* transcription via interacting with EZH2

**Figure 5:**
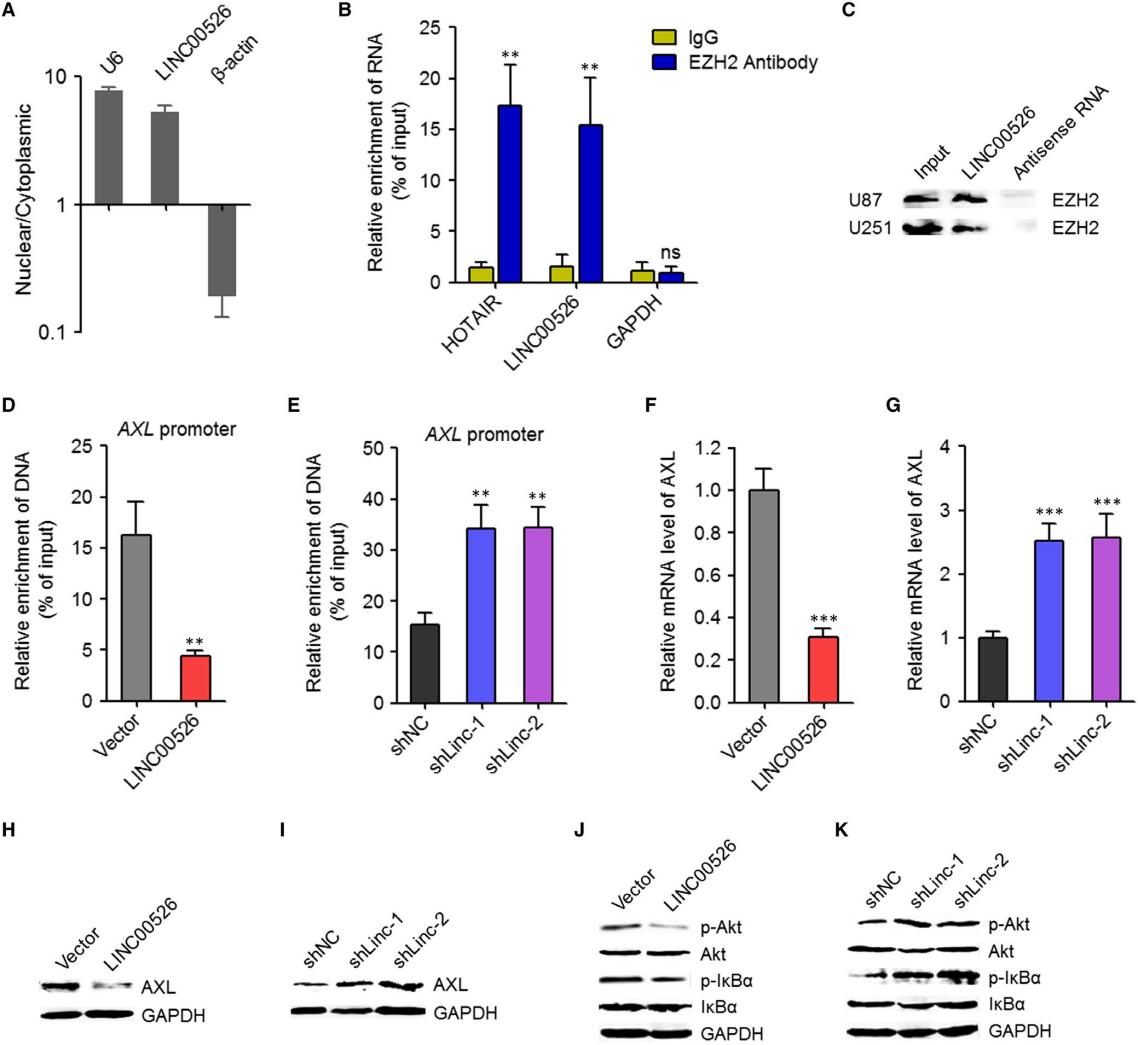
LINC00526 repressed *AXL* transcription via interacting with EZH2. A, LINC00526 subcellular location was detected by cytoplasmic and nuclear RNA purification, followed by qRT‐PCR. U6 and β‐actin were used as nuclear and cytoplasmic controls, respectively. B, RIP assays were performed in U87 cells using EZH2 specific antibody or nonspecific IgG. The enriched RNA was measured by qRT‐PCR. HOTAIR was used as positive control, and while GAPDH was used as negative control. C, RNA pull‐down assays were performed in U87 and U251 cells using in vitro transcribed biotin‐labelled LINC00526 or antisense RNA. The enriched proteins were detected by Western blot with EZH2 specific antibody. D, ChIP assays were performed in LINC00526 stably ectopically expressed and control U87 cells using EZH2 specific antibody. The enriched DNA was measured by qRT‐PCR and specific primers corresponding to *AXL* promoter. E, ChIP assays were performed in LINC00526 stably silenced and control U251 cells using EZH2 specific antibody. The enriched DNA was measured by qRT‐PCR and specific primers corresponding to *AXL* promoter. F, AXL mRNA expression levels in LINC00526 stably ectopically expressed and control U87 cells were measured by qRT‐PCR. G, AXL mRNA expression levels in LINC00526 stably silenced and control U251 cells were measured by qRT‐PCR. H, AXL protein expression levels in LINC00526 stably ectopically expressed and control U87 cells were measured by Western blot. I, AXL protein expression levels in LINC00526 stably silenced and control U251 cells were measured by Western blot. J, Phosphorylation levels of Akt and IκBα in LINC00526 stably ectopically expressed and control U87 cells were measured by Western blot. K, Phosphorylation levels of Akt and IκBα in LINC00526 stably silenced and control U251 cells were measured by Western blot. **P < 0.01, ***P < 0.001, ns, not significant, Student's *t* test (B, D, F) or one‐way ANOVA followed by Dunnett's multiple comparison test (E, G)

To determine the potential influences of LINC00526 on AXL, we first confirmed the subcellular location of LINC00526. The subcellular location of LINC00526 was predicted by lncLocator (http://www.csbio.sjtu.edu.cn/bioinf/lncLocator/).^38^ The results predicted that LINC00526 was located in the nucleus with a score of 0.63. Next, we performed cytoplasmic and nuclear RNA purification using U87 cells. The location of LINC00526 was measured by qRT‐PCR. The results showed that LINC00526 was mainly located in the nucleus (Figure 5A). Many nuclear lncRNAs were reported to interact with chromatin‐modifying complexes and modulate genes expression.^17^ About 20% nuclear lncRNAs were found to bind EZH2, a critical component of polycomb repressive complex 2 (PRC2).^39^ Interestingly, EZH2 was also reported to activate *AXL* transcription.^34^ Therefore, we further explore whether LINC00526 also binds EZH2 and whether LINC00526 influences *AXL* transcription via binding EZH2. RIP assays were performed in U87 cells. The results showed that LINC00526 was specifically enriched in EZH2 antibody group, which indicated the binding between LINC00526 and EZH2 (Figure 5B). Furthermore, RNA pulldown assays were performed using in vitro transcribed LINC00526 in U87 and U251 cells. As shown in Figure 5C, EZH2 was specifically enriched in LINC00526 group, which further supported the binding between LINC00526 and EZH2. Then, we investigated whether the binding between LINC00526 and EZH2 influences the binding of EZH2 on *AXL* promoter. ChIP assays using EZH2 specific antibody were performed in LINC00526 stably overexpressed and control U87 cells. As shown in Figure 5D, ectopic expression of LINC00526 decreased the binding of EZH2 on *AXL* promoter. ChIP assays using EZH2 specific antibody were also performed in LINC00526 stably silenced and control U251 cells. The results showed that LINC00526 silencing increased the binding of EZH2 on *AXL* promoter (Figure 5E). EZH2 was reported to activate *AXL* transcription in glioma cells. Next, the mRNA and protein levels of AXL in LINC00526 stably overexpressed and control U87 cells, and LINC00526 stably silenced and control U251 cells were measured by qRT‐PCR and Western blot. As shown in Figure 5F‐I, ectopic expression of LINC00526 decreased the mRNA and protein levels of AXL, and while LINC00526 silencing increased the mRNA and protein levels of AXL. As a receptor tyrosine kinase, AXL is well‐known to activate downstream PI3K/Akt/NF‐κB signalling. Next, we investigated the influences of LINC00526 on PI3K/Akt/NF‐κB signalling. As shown in Figure 5J, ectopic expression of LINC00526 decreased the phosphorylation levels of Akt and IκBα. LINC00526 silencing increased the phosphorylation levels of Akt and IκBα (Figure 5K). Taken together, these results suggested that LINC00526 bound to EZH2, decreased the binding of EZH2 to *AXL* promoter, repressed *AXL* transcription and repressed PI3K/Akt/NF‐κB signalling.

### 3.6 AXL repressed *LINC00526* transcription via activating NF‐κB signalling

**Figure 6:**
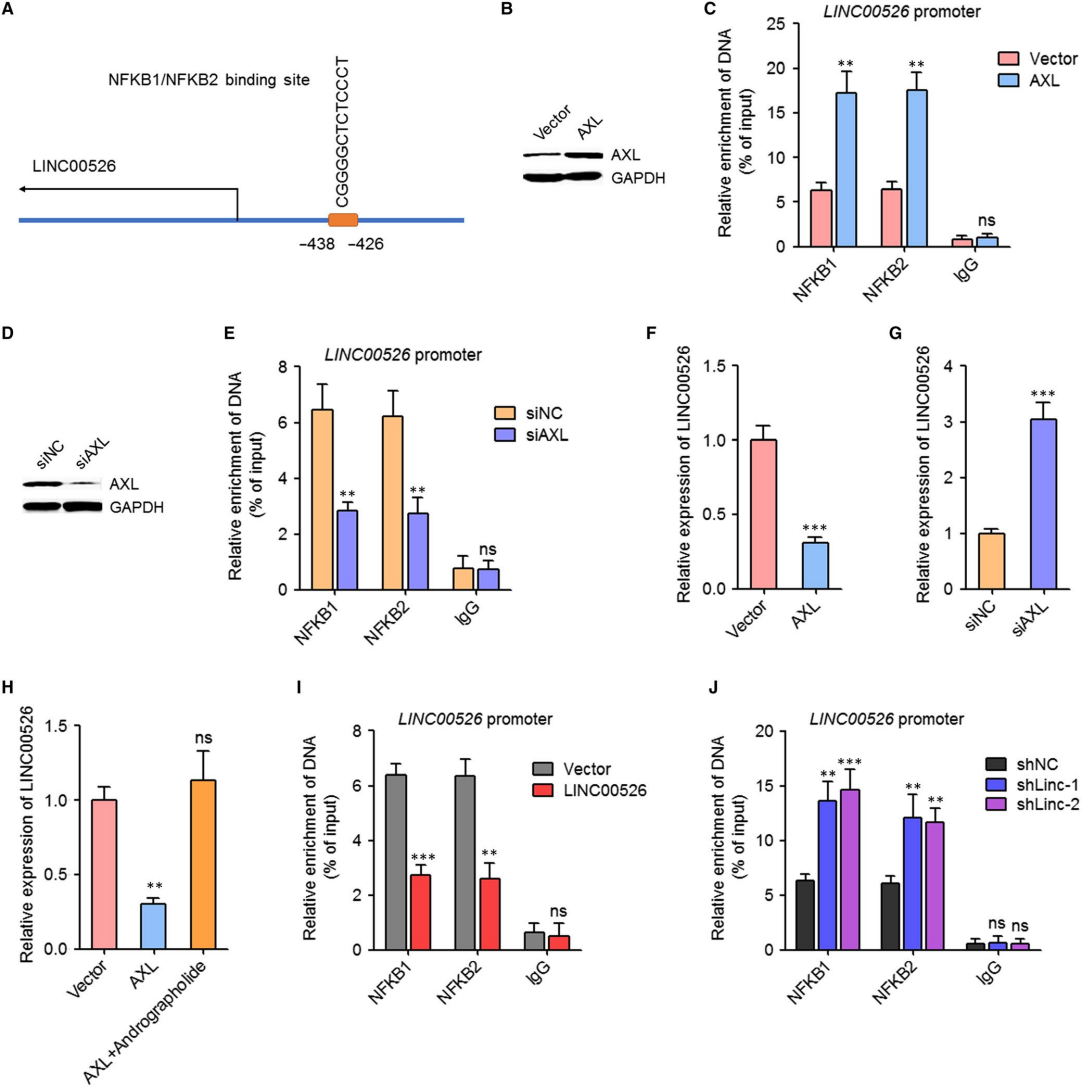
AXL repressed *LINC00526* transcription via activating NF‐κB signalling. A, Schematic outlining the predicted binding sites of NFKB1/NFKB2 on *LINC00526* promoter. B, AXL protein levels in U251 cells after transient overexpressing AXL were measured by Western blot. C, ChIP assays were performed in U251 cells after transient overexpressing AXL using NFKB1, NFKB2 specific antibody or non‐specific IgG. The enriched DNA was measured by qRT‐PCR and specific primers corresponding to *LINC00526* promoter. D, AXL protein levels in U87 cells after transient silencing AXL were measured by Western blot. E, ChIP assays were performed in U87 cells after transient silencing AXL using NFKB1, NFKB2 specific antibody or non‐specific IgG. The enriched DNA was measured by qRT‐PCR and specific primers corresponding to *LINC00526* promoter. F, LINC00526 expression levels in U251 cells after transient overexpressing AXL were measured by qRT‐PCR. G, LINC00526 expression levels in U87 cells after transient silencing AXL were measured by qRT‐PCR. H, LINC00526 expression levels in U251 cells after transient overexpressing AXL and treated with 10 μM Andrographolide for 2 days were measured by qRT‐PCR. I, ChIP assays were performed in LINC00526 stably overexpressed and control U87 cells using NFKB1, NFKB2 specific antibody or non‐specific IgG. The enriched DNA was measured by qRT‐PCR and specific primers corresponding to *LINC00526* promoter. J, ChIP assays were performed in LINC00526 stably silenced and control U251 cells using NFKB1, NFKB2 specific antibody or non‐specific IgG. The enriched DNA was measured by qRT‐PCR and specific primers corresponding to *LINC00526* promoter. **P < 0.01, ***P < 0.001, ns, not significant, Student's *t* test (C, E, F, G, I) or one‐way ANOVA followed by Dunnett's multiple comparison test (H, J)

Due to the significant inverse correlation between the expression of AXL and LINC00526, we next explored whether AXL modulates the expression of LINC00526. Intriguingly, analysing the promoter region of LINC00526 using JASPAR (http://jaspar.genereg.net/),^40^ we found one NFKB1/NFKB2 binding site at −438‐−426 upstream of the transcription start site of *LINC00526* (Figure 6A). After transient overexpressing AXL in U251 cells (Figure 6B), ChIP assays were performed using NFKB1 and NFKB2 specific antibodies. As shown in Figure 6C, NFKB1 and NFKB2 specifically bound to *LINC00526* promoter. Furthermore, ectopic expression of AXL significantly promoted the binding of NFKB1 and NFKB2 to *LINC00526* promoter (Figure 6C). After transient silencing AXL in U87 cells (Figure 6D), ChIP assays were performed using NFKB1 and NFKB2 specific antibodies. As shown in Figure 6E, NFKB1 and NFKB2 also specifically bound to *LINC00526* promoter in U87 cells. Furthermore, AXL silencing significantly repressed the binding of NFKB1 and NFKB2 to *LINC00526* promoter (Figure 6E). The influences of AXL on the transcription level of LINC00526 were investigated by qRT‐PCR. The results showed that ectopic expression of AXL significantly repressed the transcription of *LINC00526*, and while AXL silencing significantly promoted the transcription of *LINC00526* (Figure 6F,G). To confirm whether the repression of *LINC00526* transcription by AXL was mediated by the activation of NF‐κB signalling, the AXL overexpressed U251 cells were treated with NF‐κB inhibitor Andrographolide. As shown in Figure 6H, treatment with Andrographolide abolished the repressive roles of AXL on *LINC00526* transcription. Thus, these results suggested that AXL repressed *LINC00526* transcription via enhancing NF‐κB signalling. Next, we investigated whether LINC00526 regulates the effects of NF‐κB on LINC00526 via repressing AXL. ChIP assays were performed using NFKB1 and NFKB2 specific antibodies in LINC00526 stably overexpressed and control U87 cells, and LINC00526 stably silenced and control U251 cells. As shown in Figure 6I,J, in contrast to AXL, ectopic expression of LINC00526 significantly repressed the binding of NFKB1 and NFKB2 to *LINC00526* promoter, and while LINC00526 silencing significantly promoted the binding of NFKB1 and NFKB2 to *LINC00526* promoter. These results supported the auto‐regulatory roles of LINC00526 on itself.

### 3.7 The tumour suppressive roles of LINC00526 in glioma were dependent on AXL

**Figure 7:**
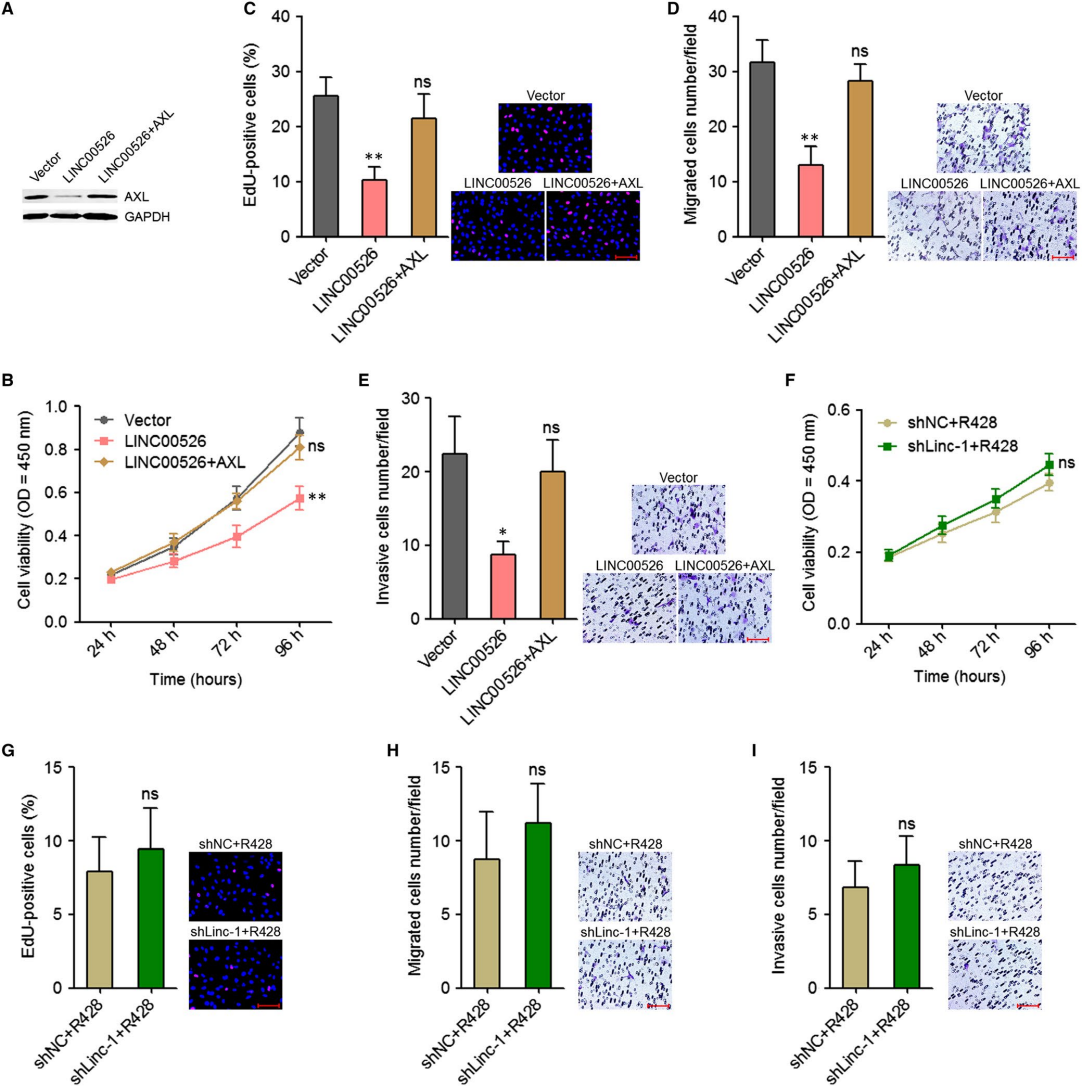
The tumour suppressive roles of LINC00526 in glioma were dependent on AXL. A, AXL protein levels in LINC00526 and AXL concurrently stably ectopically expressed and control U87 cells were measured by Western blot. B, Cell proliferation rates of LINC00526 and AXL concurrently stably ectopically expressed and control U87 cells were measured by CCK‐8 assays. OD values in 450 nm were detected to indicate cell proliferation. C, Cell proliferation of LINC00526 and AXL concurrently stably ectopically expressed and control U87 cells were measured by EdU incorporation assays. Scale bars, 100 µm. D, Cell migration of LINC00526 and AXL concurrently stably ectopically expressed and control U87 cells were measured by transwell migration assays. Scale bars, 100 µm. E, Cell invasion of LINC00526 and AXL concurrently stably ectopically expressed and control U87 cells were measured by transwell invasion assays. Scale bars, 100 µm. F, Cell proliferation rates of LINC00526 stably silenced and control U87 cells treated with 1 μM R428 were measured by CCK‐8 assays. OD values in 450 nm were detected to indicate cell proliferation. G, Cell proliferation of LINC00526 stably silenced and control U87 cells treated with 1 μM R428 were measured by EdU incorporation assays. Scale bars, 100 µm. D, Cell migration of LINC00526 stably silenced and control U87 cells treated with 1 μM R428 were measured by transwell migration assays. Scale bars, 100 µm. E, Cell invasion of LINC00526 stably silenced and control U87 cells treated with 1 μM R428 were measured by transwell invasion assays. Scale bars, 100 µm. Results are shown as mean ± SD of three independent experiments. *P < 0.05, **P < 0.01, ns, not significant, one‐way ANOVA followed by Dunnett's multiple comparison test (B‐E) or Student's *t* test (F‐I)

The above results showed that LINC00526 interacted with EZH2, repressed *AXL* transcription, and repressed PI3K/Akt/NF‐κB signalling. AXL activated NF‐κB signalling and repressed *LINC00526* transcription. Therefore, LINC00526 and AXL formed a double negative feedback loop. Next, we investigated whether the tumour suppressive roles of LINC00526 in glioma are dependent on AXL. We stably ectopically expressed AXL in LINC00526 stably overexpressed U87 cells (Figure 7A). CCK‐8 assays revealed that ectopic expression of AXL abolished the decrease in proliferation rate caused by LINC00526 overexpression (Figure 7B). Moreover, EdU incorporation assays further supported the roles of ectopic expression of AXL in the reverse of the proliferation decrease caused by LINC00526 overexpression (Figure 7C). Transwell migration and invasion assays revealed that ectopic expression of AXL abolished the decrease in migration and invasion caused by LINC00526 overexpression (Figure 7D,7). In addition, we treated LINC00526 stably silenced and control U87 cells with AXL inhibitor R428. CCK‐8 assays revealed that treatment with R428 blocked the increase in proliferation rate caused by LINC00526 silencing (Figure 7F). Moreover, EdU incorporation assays further showed that treatment with R428 blocked the pro‐proliferatory roles of LINC00526 silencing (Figure 7G). Transwell migration and invasion assays revealed that treatment with R428 blocked the increase in migration and invasion caused by LINC00526 silencing (Figure 7H,I). Thus, these results indicated that the roles of LINC00526 in the proliferation, migration and invasion of glioma cells are dependent on the regulation of AXL.

## 4 DISCUSSION

In this study, we identified a novel lncRNA LINC00526, which is down‐regulated and functions as a tumour suppressor in glioma. We first confirmed the significant down‐regulation of LINC00526 in human glioma tissues. LINC00526 expression levels were inversely correlated with aggravation of glioma. Second, the correlation between LINC00526 expression level and prognosis of glioma patients was analysed using TCGA data. The TCGA data revealed that low expression of LINC00526 is associated with poor survival of glioma patients. Gain‐of‐function and loss‐of‐function assays demonstrated that ectopic expression of LINC00526 inhibited glioma cell proliferation, migration and invasion. Reciprocally, LINC00526 silencing promoted glioma cell proliferation, migration and invasion. Therefore, our data identified LINC00526 as a potential prognostic biomarker for glioma and suggested that ectopic expression of LINC00526 may be a potential therapeutic strategy for glioma. LncRNAs GAS5, NEAT1, GACAT3, CASC9, HMMR‐AS1, LINC00152, HOTAIRM1, AC003092.1, HOXD‐AS1, OIP5‐AS1, DANCR and so on have been reported to play oncogenic or tumour suppressive roles in glioma.^32^, ^33^, ^41^, ^42^, ^43^, ^44^, ^45^, ^46^, ^47^, ^48^, ^49^ Our study further proved the critical roles of lncRNAs in glioma and provided another candidate lncRNA for glioma prognosis and therapy. Multi‐centre analyses of the correlation between LINC00526 expression and prognosis of glioma patients would further detect the potential of LINC00526 as a prognostic biomarker, which needs further investigation.

Although the expression and roles of several lncRNAs in glioma have been sufficiently investigated, the molecular mechanisms mediating the roles of lncRNAs and the reasons contributing to the dysregulation of lncRNAs in glioma are relative less investigated. In this study, we further explored the molecular mechanisms mediating the down‐regulation and tumour suppressive roles of LINC00526 in glioma. Through searching TCGA data, we found that the expression of AXL was significantly inversely associated with that of LINC00526 in glioma tissues. AXL is a well‐known receptor tyrosine kinase.^37^ Several reports have revealed that AXL is overexpressed in human glioma and predicts poor prognosis of glioma patients.^50^, ^51^ AXL was also reported to promote glioma growth, migration, invasion, tumorigenesis and primary resistance to EGFR inhibition.^37^, ^52^, ^53^ The inverse expression patterns and inverse biological roles between LINC00526 and AXL implied that whether there is negative modulation between LINC00526 and AXL.

The molecular mechanisms mediating the roles of lncRNAs are complex and diverse.^25^, ^28^ As a class of regulatory RNAs, lncRNAs directly bind proteins, DNAs, mRNAs and/or microRNAs.^35^ Through interacting with other molecules, lncRNAs regulate the expression, location and/or functions of the interacting partners.^35^ In this study, we identified that LINC00526 directly interacted with EZH2, which is a histone lysine methyltransferase and epigenetically modulates target genes expression.^54^, ^55^ In a previous report, Ott et al showed that EZH2 induces *AXL* transcription.^34^ In this study, we further found that, the binding between LINC00526 and EZH2 decreased the binding of EZH2 to *AXL* promoter, and further decreased the transcription of *AXL*. Therefore, LINC00526 repressed *AXL* expression via occupying EZH2. Via repressing AXL, LINC00526 further repressed PI3K/Akt/NF‐κB signalling. Recuse assays further found that the tumour suppressive roles of LINC00526 were dependent on the repression of AXL.

Intriguingly, we further identified LINC00526 as a target of NF‐κB signalling. We identified a NFKB1/NFKB2 binding site at the promoter of *LINC00526*, and demonstrated that NF‐κB repressed *LINC00526* transcription. Via activating NF‐κB signalling, AXL promoted the binding of NFKB1/NFKB2 to *LINC00526* promoter and repressed *LINC00526* transcription. Therefore, LINC00526 and AXL form a double negative feedback loop, which further promotes the down‐regulation of LINC00526 and the up‐regulation of AXL in glioma tissues. Several feedback regulatory loops have been identified in cancers.^13^, ^56^ The feedback regulatory loops could enlarge their modulatory roles in control gene expression and biological effects in cancers.^13^, ^56^ Because AXL/PI3K/Akt/NF‐κB signalling also regulates other genes, the effects of LINC00526 on these targets need further investigation.

In summary, this study identified a novel lncRNA LINC00526, which is lowly expressed in glioma. The down‐regulation of LINC00526 was correlated with aggravation and poor prognosis. LINC00526 inhibited glioma cell proliferation, migration and invasion via the LINC00526/EZH2/AXL/NF‐κB/LINC00526 feedback loop. Thus, this study identified LINC00526 as a potential prognostic biomarker and a candidate for therapy in glioma.

## CONFLICT OF INTEREST

The authors declare that they have no conflict of interest.

## Data Availability

The data that support the findings of this study are available from the corresponding author upon reasonable request.
